# Efficacy of SRM-IV Vestibular Function Diagnosis and Treatment System in Treating Benign Paroxysmal Positional Vertigo

**Published:** 2018-05

**Authors:** Nuan WANG, Hao ZHOU, Hongli HUANG, Deqin GENG, Xiuhua YANG, Chunyu YU, Di SHI

**Affiliations:** Dept. of Neurology, The First People’s Hospital of Xuzhou, Xuzhou, Jiangsu 221000, P.R. China

**Keywords:** SRM-IV vestibular function, Benign paroxysmal positional vertigo, Automatic repositioning

## Abstract

**Background:**

We determined the diagnostic and therapeutic effects of SRM-IV vestibular function diagnosis and treatment system on benign paroxysmal positional vertigo (BPPV).

**Methods:**

Overall, 120 patients with BPPV diagnosed in the outpatient and in-patient departments of the Vertigo Treatment Center of the First People’s Hospital of Xuzhou from January 2013 to December 2015 were selected for this study. They were randomly divided into three groups. Automatic repositioning procedure was conducted for 40 patients in the equipment repositioning group by SRM-IV vestibular function diagnosis and treatment system, conventional manual repositioning procedure was used for 40 patients in the manual repositioning group, and combination of treatment drugs (alprostadil and safflower injection) with acclimatization training was adopted in 40 patients in the drug therapy group.

**Results:**

After 1 week of treatment, the cure rate and total effective rate in the equipment repositioning group and the manual repositioning group were significantly higher than those in drug therapy group (*P*<0.05). The total effective rate was 100.0% in the equipment-repositioning group and 92.5% in manual repositioning group; the difference between the two groups was not statistically significant. The success rate of one-time treatment of anterior semicircular canal BPPV, posterior semicircular canal BPPV and lateral semicircular canal BPPV in the equipment-repositioning group were higher than those in the manual repositioning group were.

**Conclusion:**

The SRM-IV vestibular function diagnosis and treatment system are helpful in achieving effective and standard diagnosis and treatment of BPPV.

## Introduction

Benign paroxysmal positional vertigo (BPPV) is a common type of vertigo with a high incidence rate, and accounts for 17–20% of peripheral vertigo. BPPV, however, is often misdiagnosed as cerebrovascular vertigo and cervical vertigo, and unnecessary economic and psychological burdens are added to patients if inappropriate examination and treatment are administered. Canalith repositioning procedure can alleviate patients’ symptoms rapidly and relieve the pain of vertigo patients.

Currently, the manual repositioning procedures consist of Barbecue rotation therapy, Epley procedure, Semont procedure and Gufoni procedure, but the clinical efficacy of each treatment is not stable as there are problems in the manipulative standards and physician’s experience in clinical practices (1). SRM-IV vestibular function diagnosis and treatment system is currently the most advanced BPPV diagnosis and repositioning equipment in the world. It can film the performance of a patient’s eye movement during different degrees of rotation and stimulation and display it on a computer screen. Meanwhile, the direction, type, speed and intensity of nystagmus can be digitized by the equipment and then be conveyed to the physician in the most intuitive way, allowing the medical staff to make judgments on the diagnosis, treatment and efficacy.

Our clinical study utilized automatic SRM-IV vestibular function diagnosis and treatment system for diagnosis and repositioning treatment on BPPV patients as well as the conventional manual repositioning procedure for comparison analysis, in order to explore effective therapeutic methods for BPPV.

## Methods

### Objects of study

Overall, 120 patients with BPPV that were diagnosed in the outpatient and in-patient departments of the Vertigo Treatment Center of the First People’s Hospital of Xuzhou from January 2013 to December 2015 were selected for this study. All patients underwent imaging examinations including magnetic resonance imaging (MRI) or cranial computed tomography (CT) in order to exclude new cerebral infarction and cerebral hemorrhage. Patients also received routine neuro-otology tests to exclude vertigo caused by other problems in the nervous system and labyrinth.

This study was approved by the Ethics Committee of the First People’s Hospital of Xuzhou. The informed consent was signed by all patients and their families.

The patients were divided into 3 groups by the random number table. Automatic repositioning procedure was conducted for 40 patients in equipment repositioning group by SRM-IV vestibular function diagnosis and treatment system.

Manual repositioning procedure was used for 40 patients in the manual repositioning group. Lastly, treatment-combining drugs with acclimatization training was administered to 40 patients in drug therapy group.

In the equipment repositioning group, there were 13 males and 27 females aged 24–80 years old, with an average age of 45.21±12.36 years old. The duration of disease was ranged between 15 days and 8 years, with an average duration of 2.23±3.61 years. In manual repositioning group, there were 14 men and 26 women aged 23–76 years old, with an average age of 44.83±12.50 years old. The duration of disease was ranged between 4 days and 9 years, with an average duration of 2.15±3.07 years. In the drug therapy group, there were 15 males and 25 females aged 21–79 years old, with an average age of 43.16±12.44 years old. The duration of disease was between 11 days and 7 years, with an average duration of 2.22±3.13 years. The statistical processing of the general data in two groups of patients confirmed that the differences were not significant (*P*>0.05) and were therefore comparable.

### Diagnostic criteria

All patients met the following BPPV diagnostic criteria published by the Chinese Otorhinolaryngology Head Surgery Society of the Chinese Medical Association in 2007 (2): patients with history of transient vertigo when the head was moved to a specified position; patients with positive positioning nystagmus test (positive Dix-Hallpike test indicated posterior or anterior semicircular canal BPPV, and positive roll test indicated lateral semicircular canal BPPV), and with short latent period (<30s) and fatigue. Nystagmus features of posterior semicircular canal BPPV. When the affected ear was directed towards the ground, vertical torsion nystagmus (vertical component towards the upper pole of the eyeball and torsion component towards the ground) marked by the upper pole of the eyeball occurred. When the patient returned to the sitting position, the directions of nystagmus were reversed. The duration of nystagmus in canalithasis was <1min and that of nystagmus in cupulolithiasis was ≥1min. The nystagmus features of anterior semicircular canal BPPV were as follows. When the affected ear was directed toward the ground, vertical torsion nystagmus (vertical component towards the lower pole of eyeball and torsion component towards the ground) marked by the upper pole of the eyeball occurred. When the patient returned to sitting position, the directions of nystagmus were reversed. The duration of nystagmus in canalithasis was <1min and that of nystagmus in cupulolithiasis was ≥1min. Nystagmus features of the lateral semicircular canal BPPV were as follows. Canalithasis could induce geotropic horizontal nystagmus in the bilateral positioning test, and the duration of nystagmus was <1min. The cupulolithiasis could induce apogeotropic horizontal nystagmus in the bilateral positioning test, and the duration of nystagmus was ≥1min.

### BPPV diagnosis and treatment system

The system was composed of the hardware system, software system and background workstation. The hardware system consisted of an automatic three-dimensional rotatory chair as well as a wireless video capturing and acquisition device for eye movement. The automatic three-dimensional rotatory chair could rotate 360° or above in a three-dimensional space under the control of computer software, of which the maximum velocity could reach 180°/s and the maximum acceleration, was 200°/s^2^. The eye movement was captured using the wireless video goggle and then displayed on the liquid crystal display, so that the position and movement of the eyeball could be clearly observed. Software system, that is, computer software programs for diagnosis and treatment of BPPV, was composed of the vestibular function examinations such as temperature test, rotation test, etc. The result analysis program, storage and analysis program for video eye movement and control program for the rotation angle and speed of three-dimensional rotatory chair. Background workstation could formulate inspection and treatment schemes for BPPV patients, including display windows for video eye movement, nystagmus curve and rotatory chair position as well as display window for three-dimensional anatomic images of the bilateral cochlea and vestibular system that changed with the movement of rotatory chair. As a result, the variation of the above-mentioned parameters of the patients during the movement could be displayed in real time.

### Therapeutic methods

Infrared video goggle was used in the manual repositioning group, and the roll test and Dix-Hallpike test were accomplished on the examining table. The Barbecue rotation therapy was used for patients diagnosed with lateral semicircular canal BPPV, Epley canalith repositioning procedure was applied to patients with posterior semicircular canal BPPV, and Semont canalith repositioning procedure was utilized for patients with anterior semicircular canal BPPV. The rreatment combining alprostadil (Manufacturer: Benxi Hygecon Pharmaceutical Co., Ltd., Approval number: NMPN H20093175) and safflower injection (Manufacturer: Ya’n Sanjiu Medical & Pharmaceutical Co., Ltd., Approval number: NMPN Z51020673) with acclimatization training was administered in the drug therapy group. Alprostadil (10 μg) was mixed with 100mL normal saline and then infused intravenously once a day. Aafflower injection (20mL) was mixed with 250mL normal saline and then infused intravenously once a day. Seven consecutive days of drug administration were taken as a course of treatment. The acclimatization training could reduce the symptoms induced by special movement by means of repeated head and visual movement.

SRM-IV vestibular function diagnosis and treatment system was utilized in the equipment-repositioning group, and all patients accomplished the roll test and Dix-Hallpike test on the system. Anterior semicircular canal BPPV: The patient was seated in the straight-ahead position and the whole body was turned left or right 45° and remained for 1second. Then, it leaned forward 120° within 1.5s to observe the disappearing of nystagmus and vertigo. After that, the body was turned 110° and remained there for 60s to observe the disappearance of nystagmus and vertigo. The body was continuously turned 130°, back to the starting position. Posterior semicircular canal BPPV: The patient was seated in the straight position, and the whole body was turned left or right 45° and remained there for 1second. Then, it was leant backward 120° within 1.5 s to observe the disappearance of nystagmus and vertigo. After that, the body was turned 110° and remained for 60s to observe the disappearing of nystagmus and vertigo. Then, the body was further turned 130°, back to the starting position. Lateral semicircular canal BPPV (cupulolithiasis type) of the right side: The patient was seated in the straight position and then slowly rotated to the supine position with the head risen 30°. The patient turning to the left 90° was taken as the starting position of repositioning procedure. The whole body was turned to the right 90° within 1.5 seconds and remained for there for 60s to observe the disappearance of nystagmus and vertigo. Again, it was turned to the right 90° within 1.5s and remained there for 60s to observe the disappearance of nystagmus and vertigo. Then, the body was turned to the right 90° and remained there for 60s to observe the disappearance of nystagmus. After that, the patients were further turned to the right 90° and remained there for 60s to observe the disappearance of nystagmus, and then were finally back to the starting position. Lateral semicircular canal BPPV (cupulolithiasis type) of the left side: the patient was seated in the straight position and then leaned towards the left side rapidly for 90° within 1.5 seconds and remained for about 180s to observe the disappearance of nystagmus and vertigo. Then, the body was turned to the right for 90° C within 1.5 s and remained for there for 60 s to observe the disappearance of nystagmus and vertigo, and then the patients were returned to the starting position. The process was repeated twice.

### Observation indexes

The patients in the equipment repositioning group and the manual repositioning group were reexamined 48 hours after treatment, in which roll test or the Dix-Hallpike test was performed again to determine whether there was nystagmus and vertigo in patients. The second repositioning treatment was carried out on patients with positive provocative tests, further consultation was conducted after 1-week of observation, and the clinical efficacy was assessed. If symptoms were relieved in drug therapy group 1 week after treatment combining drug administration with acclimatization training, the one-time treatment was successful.

### Evaluation criteria of therapeutic effects

The evaluation of therapeutic effects was based on the clinical efficacy criteria in the *Diagnosis Basis and Curative Effect Appraisal of Benign Paroxysmal Positional Vertigo (2006, Guiyang)* formulated by the Chinese Otorhinolaryngology Head Surgery Society of Chinese Medical Association in 2007 (2). Recovery indicated that the symptoms of positional nystagmus and vertigo disappeared completely. Effective indicated that the symptoms of positional nystagmus and vertigo were relieved significantly. Ineffective indicated that the symptoms of positional nystagmus and vertigo were not improved remarkably, or were even aggravated or converted to other types of BPPV. The sum of the cure rate and effective rate was used as the total effective rate.

### Statistical methods

SPSS 15.0 software (Chicago, IL, USA) was used for data processing, and chi-square test was applied to the comparisons of indexes. *P*<0.05 suggested that the difference was statistically significant.

## Results

### Comparisons of clinical efficacy

The statistical results of clinical efficacy in three groups of patients after 1 week of treatment are shown in Table 1. The cure rates and total effective rates in the equipment repositioning group and manual repositioning group were significantly higher than those in drug therapy group (*P*<0.05). There were no significant differences between the equipment repositioning group and the manual repositioning group (*P*>0.01).

**Table 1:** Comparisons of clinical efficacy in three groups after 1 week of treatment [n (%)]

***Group***	***Case***	***Cured***	***Effective***	***Ineffective***	***Total effective rate***
Equipment repositioning group	40	37 (92.5)^*^ *^#^*	3 (7.5)	0 (0.0)	40 (100.0)^*^ *^#^*
Manual repositioning group	40	34 (85.0)^*^	3 (7.5)	3 (7.5)	37 (92.5)^*^
Drug therapy group	40	9 (22.5)	11 (27.5)	20 (50.0)	20 (50.0)

Note:

* indicates comparisons with drug therapy group, P<0.05;

# indicates comparisons with manual repositioning group, P>0.05

Upon reexamination 1 week later, patients in the manual repositioning group, who were treated by the equipment repositioning procedure, exhibited that they were cured.

### Comparisons of treatment on different types of BPPV

In the equipment-repositioning group, there were 5 cases of anterior semicircular canal BPPV, 22 cases of posterior semicircular canal BPPV and 13 cases of lateral semicircular canal BPPV. In the manual repositioning group, the number of cases of anterior, posterior and lateral semicircular canal BPPV was 4, 23 and 13, respectively. The success rates of one-time treatment of different types of BPPV in the two groups are shown in Table 2. There was no significantly difference between the equipment repositioning group and manual repositioning group (*P*>0.001), however, the success rates of one-time treatment in the equipment repositioning group were higher than those in manual repositioning group were.

**Table 2:** Comparisons of success rates of one-time treatment of different types of BPPV in the two groups (%)

***Group***	***Anterior semicircular canal BPPV***	***Posterior semicircular canal BPPV***	***Lateral semicircular canal BPPV***
Equipment repositioning group	80.00 (4/5)^*^	90.90 (20/22)^*^	84.61 (11/13)^*^
Manual repositioning group	75.00 (3/4)	86.96 (20/23)	76.92 (10/13)

Note:

* indicates comparisons with manual repositioning group, P>0.05

## Discussion

BPPV is one of the most common peripheral vestibular disorders with complains of vertigo, which was first discovered by the physiologist Bárány in 1921 (3). The specific clinical features and posture test methods were determined by Dix and Hallpike in 1952 (4). The pathogenic mechanism of BPPV was identified during subsequent development and perfection. According to the different types of semicircular canals involved, BPPV can be divided into three types, namely, the posterior semicircular canal BPPV, anterior semicircular canal BPPV and lateral semicircular canal BPPV. Studies have shown that the posterior semicircular canal BPPV is the most typical, and is presumed that the disease is related to the anatomical position of posterior semicircular canal and vestibule when the patient is in the upright position. It is the most likely to occur when the otolith fragments are displaced (5). According to our study, patients with posterior semicircular canal BPPV account for the largest proportion in both groups, followed by lateral semi-circular canal BPPV and anterior semicircular canal BPPV, which was corroborated with results from other studies. The major diagnostic method for BPPV is the positioning test, and the major treatment methods is the manual repositioning procedure, also known as canalith repositioning procedure (CRP). This treatment is conducted by restoring the position of the displaced otolith by adjusting the patient’s posture. However, there are differences in manual repositioning procedure and diagnosis among patients as the operating methods are different during treatment and the clinical experience of physicians is variable (6).

There are three pairs of semicircular canals in the human body located in three planes, which are relatively perpendicular to each other. The automatic SRM-IV vestibular function diagnosis and treatment system can maximize stimulation on the semicircular canal by corresponding the movement mode of the plane in which the semi-circular canal is located. Adequate stimulation on the semicircular canal may lead to the occurrence of positioning nystagmus, and the direction of nystagmus has a corresponding relationship with the stimulated semicircular canal (7, 8). The automatic SRM-IV vestibular function diagnosis and treatment system can be utilized to observe eye movement using video goggles and effectively identify the direction of nystagmus, thus making a definite diagnosis on the problematic semi-circular canal. As the spatial positions of semicircular canal and head stay fixed, in the process of adjusting patient’s posture, the position of the otolith can be changed by gravity, which allows the displaced otolith back to the utricle again (9). The automatic SRM-IV vestibular function diagnosis and treatment system can complete the rotation actions in a three-dimensional plane, stimulate the problematic semicircular canal, and display the incurred positioning nystagmus on the computer screen. After the problematic semicircular canal is diagnosed, the system can adjust the posture of the fixed patient, alter the three-dimensional position of the semicircular canal and accomplish the repositioning procedure by the effect of gravity. Using the automatic SRM-IV vestibular function diagnosis and treatment system to diagnose the BPPV can reduce difficulties in changing the patient’s posture for different reasons, including fear, obesity, poor body-adjustment capacity and limited cervical activity (10).

In the conventional manual repositioning procedure, problems associated with changing body position can easily to occur, positioning nystagmus cannot be guided in the patient, and a false negative state may easily to happen (11). Meanwhile, by means of automatic SRM-IV vestibular function diagnosis and treatment system, the patient’s body is fixed in a chair so that the whole body’s position can be changed at the same time. Moreover, eye movement of the patient can be observed through video goggles, and the repositioning effects can be displayed directly. It has high repeatability and can reduce the involvement of cervical activity, and therefore, is accessible to patients that are complicated with cervical spine diseases and cannot be treated with manual repositioning procedure. Furthermore, the diagnosis and treatment system is less difficult to operate, and thus, it effectively avoids the impact on the ultimate therapeutic effects due to low proficiency and accuracy of manipulation (12).

We observed through our study that, after 1 week of treatment, the total effective rates in both the equipment repositioning group and the manual repositioning group were above 80%, and the cure rates and total effective rates in both groups were significantly higher than those in the drug therapy group, which proves that both automatic SRM-IV vestibular function diagnosis, treatment system and manual repositioning procedure can effectively alleviate the symptoms of positioning nystagmus and vertigo in patients with BPPV. However, the cure rate and total effective rate in the equipment-repositioning group were higher than those in manual repositioning group were. In addition, patients in the manual repositioning group that were treated by the equipment repositioning upon reexamination 1 week later exhibited that the manual repositioning procedure was ineffective, but were cured by equipment repositioning. We also demonstrated in Table 2 that, concerning different types of BPPV, the success rate of one-time treatment in the equipment-repositioning group was higher than those in the manual repositioning group were. Furthermore, the course of treatment in the equipment-repositioning group is shorter than that in manual repositioning group, thus there are some advantages in this aspect.

## Conclusion

The automatic SRM-IV vestibular function diagnosis and treatment system has realized the full automation and standardization of conventional manual induction and repositioning procedure in terms of localization, quantification and speed fixing, so that 100% of therapeutic effect can be achieved. Therefore, it can provide an accurate basis for clinical diagnosis and treatment of BPPV and can implement standard operations. Thus, it is suitable for popularization and application in clinical practices.

## Ethical considerations

Ethical issues (Including plagiarism, informed consent, misconduct, data fabrication and/or falsification, double publication and/or submission, redundancy, etc.) have been completely observed by the authors.

## References

[1] Tegeler L Blumer J (2018). OMT May Be Helpful in the Management of Benign Paroxysmal Positional Vertigo. J Am Osteopath Assoc, 118(1): 51–52. 29309095 10.7556/jaoa.2018.010

[2] Rosenfeld RM Shiffman RN (2009). Clinical practice guideline development manual: a quality-driven approach for translating evidence into action. Otolaryngol Head Neck Surg, 140(6): S1–43. 19464525 10.1016/j.otohns.2009.04.015 PMC2851142

[3] Aron M Bance M (2013). Insights into horizontal canal benign paroxysmal positional vertigo from a human case report. Laryngoscope, 123(12): 3197–3200. 23775485 10.1002/lary.24260

[4] Lbekwe TS Rogers C (2012). Clinical evaluation of posterior canal benign paroxysmal positional vertigo. Niger Med J, 53(2): 94–101. 23271854 10.4103/0300-1652.103550 PMC3530256

[5] Wei X (2016). Progress in diagnosis and treatment of benign paroxysmal positional vertigo. Lin Chung Er Bi Yan Hou Tou Jing Wai Ke Za Zhi, 30(5): 345–348. 27382671

[6] Lin GC Basura GJ Wong HT Heidenreich KD (2012). Canal switch after canalith repositioning procedure for benign paroxysmal positional vertigo. Laryngoscope, 122(9): 2076–2078. 22549695 10.1002/lary.23315

[7] Wen C Chen T Chen F Liu Q Li S Cheng Y Lin P (2014). Investigation of the reverse phase nystagmus in positioning test for benign paroxysmal positional vertigo. Zhonghua Er Bi Yan Hou Tou Jing Wai Ke Za Zhi, 49(5): 384–389 [In Chinese]. 25017222

[8] Chen X Li P Gu X Lin S Zhang R (2011). The relevance of high stimulus rate ABR and recurrent vertigo and its clinical significance. Lin Chung Er Bi Yan Hou Tou Jing Wai Ke Za Zhi, 25(7): 289–291. 21710712

[9] Tang H Li W (2017). Advances in the diagnosis and treatment of benign paroxysmal positional vertigo. Exp Ther Med, 14(3): 2424–2430. 28962176 10.3892/etm.2017.4837 PMC5609213

[10] Nair MA Mulavara AP Bloomberg JJ Sangi-Haghpeykar H Cohen HS (2018). Visual dependence and spatial orientation in benign paroxysmal positional vertigo. J Vestib Res, 27(5–6): 279–286. 29400684 10.3233/VES-170623 PMC5801771

[11] Do YK Kim J Park CY Chung MH Moon IS Yang HS (2011). The effect of early canalith repositioning on benign paroxysmal positional vertigo on recurrence. Clin Exp Otorhinolaryngol, 4(3): 113–117. 21949575 10.3342/ceo.2011.4.3.113 PMC3173700

[12] Gu CY Han WW Wu YQ Fan ZY Chen CJ Chen HM (2018). Study on bone metabolism in postmenopausal women with idiopathic benign paroxysmal positional vertigo. Zhonghua Er Bi Yan Hou Tou Jing Wai Ke Za Zhi, 53(2): 134–137. 29429184 10.3760/cma.j.issn.1673-0860.2018.02.010

